# Clonal gene signatures predict prognosis in mesothelioma and lung adenocarcinoma

**DOI:** 10.1038/s41698-024-00531-y

**Published:** 2024-02-23

**Authors:** Yupei Lin, Bryan M. Burt, Hyun-Sung Lee, Thinh T. Nguyen, Hee-Jin Jang, Claire Lee, Wei Hong, Robert Taylor Ripley, Christopher I. Amos, Chao Cheng

**Affiliations:** 1 Department of Medicine, Baylor College of Medicine, Houston, TX 77030 USA; 2 Institute for Clinical and Translational Research, Baylor College of Medicine, Houston, TX 77030 USA; 3 The Dan L Duncan Comprehensive Cancer Center, Baylor College of Medicine, Houston, TX USA; 4 Systems Onco-Immunology Laboratory, David J. Sugarbaker Division of Thoracic Surgery, Michael E. DeBakey Department of Surgery, Baylor College of Medicine, Houston, TX 77030 USA; 5 Mesothelioma Treatment Center, Baylor St. Luke’s Medical Center, Houston, TX 77030 USA

**Keywords:** Mesothelioma, Non-small-cell lung cancer

## Abstract

Malignant pleural mesothelioma (MPM) is a rare but lethal pleural cancer with high intratumor heterogeneity (ITH). A recent study in lung adenocarcinoma has developed a clonal gene signature (ORACLE) from multiregional transcriptomic data and demonstrated high prognostic values and reproducibility. However, such a strategy has not been tested in other types of cancer with high ITH. We aimed to identify biomarkers from multi-regional data to prognostically stratify MPM patients. We generated a multiregional RNA-seq dataset for 78 tumor samples obtained from 26 MPM patients, each with one sample collected from a superior, lateral, and inferior region of the tumor. By integrating this dataset with the Cancer Genome Atlas MPM RNA-seq data, we selected 29 prognostic genes displaying high variability across different tumors but low ITH, which named PRACME (Prognostic Risk Associated Clonal Mesothelioma Expression). We evaluated PRACME in two independent MPM datasets and demonstrated its prognostic values. Patients with high signature scores are associated with poor prognosis after adjusting established clinical factors. Interestingly, the PRACME and the ORACLE signatures defined respectively from MPM and lung adenocarcinoma cross-predict prognosis between the two cancer types. Further investigation indicated that the cross-prediction ability might be explained by the high similarity between the two cancer types in their genomic regions with copy number variation, which host many clonal genes. Overall, our clonal signature PRACME provided prognostic stratification in MPM and this study emphasized the importance of multi-regional transcriptomic data for prognostic stratification based on clonal genes.

## Introduction

Malignant pleural mesothelioma (MPM) is an aggressive cancer type arising from the mesothelial cells of the pleura which lines the internal surface of chest and surrounds the lung^1^. The majority of mesothelioma cases are linked to asbestos exposure while a small number are attributable to prior chest irradiation. Genetic predisposition also contribute to this disease^2,3^. MPM tumors are divided into three histological subtypes including epithelioid, biphasic (or mixed), and sarcomatoid. Patients diagnosed with early-stage disease are amenable to curative surgery^4^. In addition to chemotherapy, immune checkpoint blockade therapies have emerged as another FDA-approved treatment option for MPM patients^5,6^. However, the development of resistance is a major concern and patients’ responses to these treatments varied dramatically. For example, almost 50% of mesothelioma patients eventually developed resistance to chemotherapy^7^ Similarly, for patients treated with dual agen immune checkpoint inhibitors in the Checkmate 743 trial, only 8% completed the two-year treatment course. As such, a critical need for biomarkers that prognostically stratify MPM patients for improving personalized treatment is urgently needed. Genomic profiling has been investigated but has not led to practical clinical applications^8,9^.

Current MPM scoring systems incorporated European Organization for Research and Treatment of Cancer (EORTC) and the Cancer and Leukemia Group B (CALGB)^10,11^. These two scoring systems included poor performance status, high white blood count, male sex, sarcomatous subtypes and a few other systemic factors. The Mesothelioma Weighted Grading Scheme (MWGS) proposed another risk-score based on BAP1 expression, histological type, age, and other factors to predict patient survival^12^. However, there currently exist no established transcriptomic gene signatures that have been developed for clinical settings. Transcriptomic profiling by microarrays and RNA-seq has been widely used to characterize tumor samples^13^. Based on the resultant gene expression data, numerous gene signatures have been developed for prognostic stratification^9,14^. However, the majority of expression-based gene signatures have low reproducibility when applied to independent data^9^. One of the critical reasons is the high transcriptomic intratumor heterogeneity (RNA-ITH) of tumors. ITH represents the genomic and transcriptomic variations among different regions within the same tumor, which is implicated in the development of treatment resistance and failure^15^.

Both MPM and lung adenocarcinoma (LUAD) are thoracic cancers, but the former arises from the mesothelial cells of the pleura along the chest and lung, while the latter often forms in the lungs. Compared with LUAD. MPM is usually more aggressive and associated with a poor prognosis regardless of the stage. Additionally, tumorigenesis is caused by different genetic alternations: in MPM loss of BAP1 and NF2 are used as biomarkers while in LUAD EGFR and TP53 are the most frequently mutated genes^16,17^. While there are substantive differences between these two thoracic cancer histologies, some of the environmental etiologies are shared and far more studies have been performed on LUAD. For example, cigarette smoke acts synergistically with asbestos to increase the risk of lung cancer in patients exposed to both carcinogens. Evaluating commonalities in risk modeling for the two diseases may provide novel insights for developing a prognostic model for MPM.

In order to overcome RNA-ITH, a previous study used multi-regional data and developed a gene signature named ORACLE (outcome risk-associated clonal lung expression) to stratify patients with non-small-cell lung cancer (NSCLC). ORACLE consists of 23 clonally expressed genes with low intratumor heterogeneity (ITH) but high intertumor heterogeneity (Q4 genes) in NSCLC^9^. These genes tend to be located in chromosomal regions with frequent clonal amplifications and their expression is driven by their copy number variations. By selecting clonal genes, ORACLE reduced the effect of sampling bias and achieved high reproducibility in the prognostic stratification of NSCLC samples. However, the effectiveness of this strategy has not been assessed in other cancer types^9^.

Similar to lung cancer, MPM has a high level of RNA-ITH^18^. To date, no prognostic signatures have been translated into clinical applications^14,19^. In this study, we applied the same strategy as ORACLE and developed a clonal gene signature for predicting patient prognosis in MPM. To this end, we generated a high-quality multi-regional RNA-seq expression dataset including 78 samples collected from 26 MPM patients, each having 3 regions (superior, lateral and inferior) of the same tumor. By combining this dataset with the TCGA-MESO RNA-seq data, we defined the clonal gene signature with 29 genes named Prognostic Risk Associated Clonal Mesothelioma Expression (PRACME). We evaluated and demonstrated the prognostic value of PRACME in multiple independent MPM datasets. Importantly, we found that PRACME, originally developed for MPM, and ORACLE, originally developed for NSCLC, can each cross-predict prognosis in the two cancer types.

## Results

### The development of a clonal gene signature for prognostic prediction in MPM

**Fig. 1:**
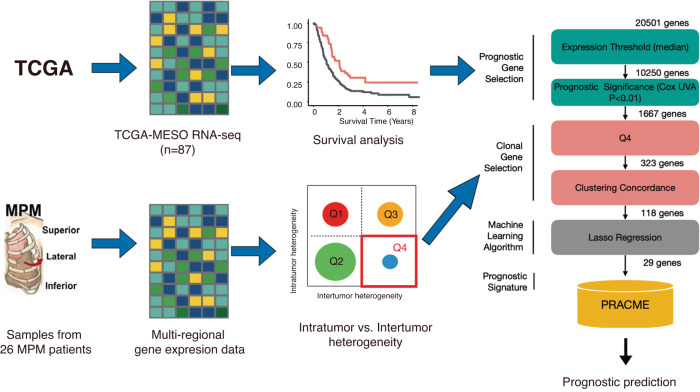
Schematic diagram in the study. (Left) Data used to define PRACME signature include The Cancer Genome Atlas mesothelioma (TCGA-MESO) and private multi-regional MPM patients data. (Right) Detailed steps of selecting final signature with color green to indicate prognostic gene selection and color pink to indicate clonal gene selection.

Based on the multiregional RNA-seq data, we calculated the intratumor and intertumor heterogeneity scores of all genes and identified 3,056 (15.07%) Q4 genes with high intertumor heterogeneity but low expression variations across the three regions with the same tumor. From these genes, we selected a subset of prognostic genes, which were identified from prognostic analysis by using the TCGA-MESO (*n* = 87). Following that, a core set of clonal prognostic genes were selected by the lasso regression model, which forms the PRACME gene signature (see Fig. 1a for detailed steps). PRACME consists of 29 genes (see Supp. Table 1), some of which are well-known in cancer development. One example is COL11A1, which has shown to be overexpressed in multiple cancers and relates to patient prognosis^20^; another example is MAP4K4, which plays a role in cancer cell proliferation and was reported to exhibit differential expression in different stages of MPM^21^.

### PRACME signature is predictive of prognosis in multiple independent MPM datasets

**Fig. 2:**
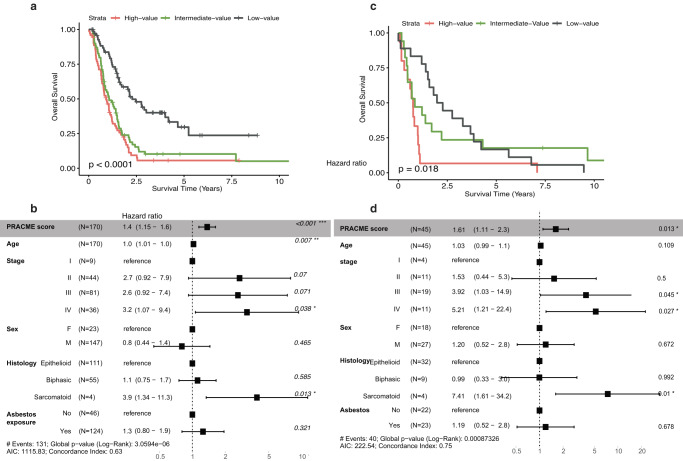
The clonal signature is predictive of patient prognosis in MPM. In Bueno MPM patients, (**a**) We stratified all patients with PRACME score into 3 subgroupings: high-, intermediate-, and low-score groups. We showed their survival difference by Kaplan–Meier plots. **b** Multivariable Cox analysis was performed with age, stage, sex, histology, and, asbestos exposure as additional clinical variables; In Bott MPM patients, (**c**) We stratified all patients with PRACME score into three subgroupings: high-, intermediate-, and low-score groups and showed their survival difference by Kaplan–Meier plots. **d** Multivariable Cox analysis was performed with age, stage, sex, and histology as additional clinical variables.

We then evaluated the prognostic value of PRACME in two independent MPM datasets. In the dataset from Bueno et al, univariable analysis indicated that the PRACME score was significantly associated with patient survival from surgery (HR > 1, *p* = 3e−06, univariable Cox regression, Supplementary Table 2). When patients were stratified into 3 groups of equal sizes, the high-score groups demonstrated significantly shorter survival time than medium-score groups or low-score groups (Fig. 2a). Multivariable Cox regression analysis indicated that PRACME score provided additional prognostic values after adjusting for established clinical variables, including age, sex, stage, histology, and asbesto exposure (Fig. 2c and Supplementary Table 2). The prognostic value of PRACME was further confirmed the dataset from Bott et al. (Fig. 2b, d). On the other hand, we found that ORACLE signature, derived from LUAD multi-regional data, was also informative in MPM patients in TCGA-MESO, Bueno and Bott datasets (Supplementary Tables 2, 3).

### Association of PRACME signature score with clinical and genomic features

**Fig. 3:**
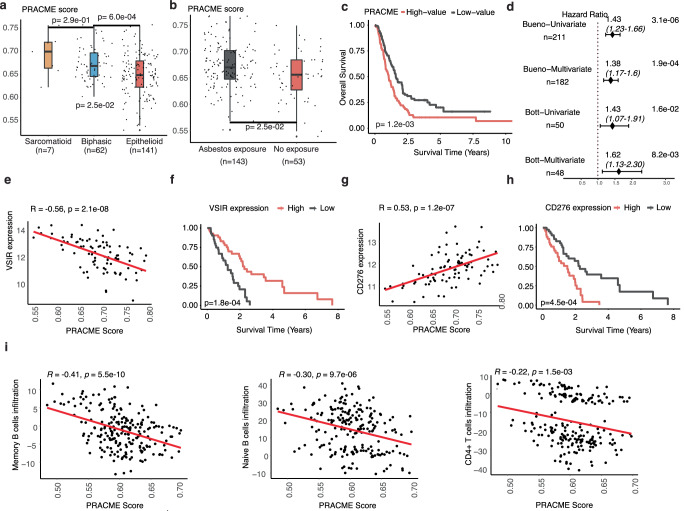
Association of PRACME signature score with clinical and genomic features. In Bueno MPM data, (**a**) PRACME score in three major histologies: epithelioid, biphasic, and sarcomatoid; *P*-value was calculated from pairwise comparison among 3 groups by using the *t* test. **b** PRACME score in subgrouping of asbestos exposure and no asbestos exposure. **c** PRACME Score Kaplan–Meier plot in epithelioid patients. **d** Forest plots showed univariable and multivariable Cox regression analysis in both Bueno and Bott datasets. **e**, **f** Correlation between PRACME score and immune gene VSIR and corresponding correlation between VSIR gene expression and patient survival. **g**, **h** Correlation between PRACME score and CD276 gene expression and corresponding correlation between CD276 gene expression and patient survival. **i** Correlation of immune infiltration of Memory B cells, Naïve B cells, and CD4+ T cells and PRACME score.

Next, we systematically examined PRACME’s prediction power under potential risk factors. Among the three main mesothelioma subtypes, including epithelioid (E), sarcomatoid (S) and biphasic (B), we found that the difference between each subtype remained significant. Further, sarcomatoid samples showed the highest median PRACME score, which correspondingly led to the poorest prognosis (Fig. 3a, Supplementary Table 4, Supplementary Fig. 1)^22^. Subgrouping patients based on asbestos exposure showed that PRACME score was significantly high in the exposed group, and patients proved to have longer survival than those who were not exposed (Fig. 3b, Supplementary Table 4, Supplementary Fig. 1). In addition, we witnessed a separation of PRACME scores for epithelioid patients, with worse prognosis for those with higher scores (Fig. 3c). We further investigated a list of molecular characteristics calculated by Thorsson et al for TCGA samples^23^. Our results indicate that MPM samples with higher PRACME scores tended to have higher proliferation rate, higher pathway activity in TGF-β response, and higher level of intratumor heterogeneity (Supplementary Table 5). We identified genes that are correlated with PRACME scores across all samples in the TCGA-MESO dataset. Enrichment analysis indicated that positively correlated genes are enriched for cell-cycle related pathways, especially related to mitosis and DNA replications (Supplementary Table 6). In contrast, negatively correlated genes are enriched in oxidations and tyrosine metabolism.

Interestingly, we found many immune-related genes are highly correlated the PRACME scores. For example, we observed a significant correlation between PRACME score and the expression level of VSIR gene (*R* = −0.56, *P* = 2.1e−08), which encoded an important immune checkpoint protein, V-type immunoglobulin domain-containing suppressor of T cell activation^24^. As a matter of fact, the expression of VSIR was significantly associated with patient prognosis in MPM (Fig. 3e, f). Another example is CD276, an immunostimulatory checkpoint gene that inhibits tumor antigen-specific immune responses^25^. CD276 had a postive correlation with PRACME score (*R* = 0.53, *P* = 1.2e−07) and its high expression led to shorter patients’ survival correspondingly (Fig. 3g, h). To further investigate the association between PRACME scores with immune cell infiltration in the tumor microenvironment, we performed computational analysis to infer the infiltration level of 6 major immune cell types based on the transcriptomic profiles of MPM samples. In both Bueno and TCGA-MESO datasets, we observed a negative correlation between PRACME score and immune infiltration, indicating samples with higher PRACME scores tended to have lower infiltration (Fig. 3i, Supplementary Fig. 3).

### PRACME signature is also predictive of prognosis in lung adenocarcinoma

**Fig. 4:**
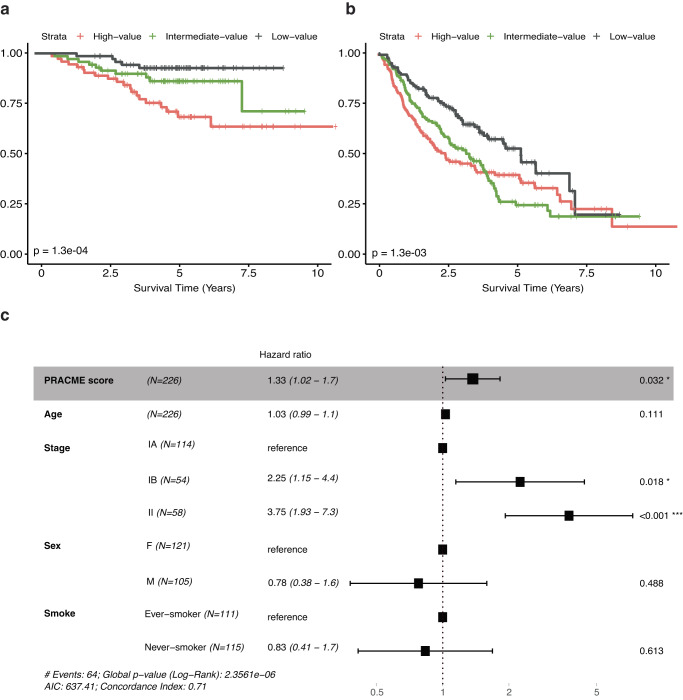
PRACME signature is also predictive of prognosis in lung adenocarcinoma. **a** In Okayama LUAD patients, PRACME score in all patients with three subgrouping: high-. intermediate- and low- score. **b** In Shedden LUAD patients, PRACME score in all patients with three subgrouping: high-. intermediate- and low- score. Kaplan–Meier plots with log-rank *P*-values calculated. **c** In Okayama LUAD patients, multivariable Cox analysis was performed with age, stage, sex, and smoking history as additional clinical variables.

Interestingly, we discovered that PRACME could also predict prognosis in LUAD, although it is developed fully based on MPM data. In the Okayama data, we found that PRACME score was associated with days before relapse (HR > 1, *p* = 6e−04). Additionally, 3 groups of patients, based on risk scores, demonstrated the low-score group had longer recurrence-free survival (Fig. 4a). Similar results were observed in Shedden LUAD dataset (Fig. 4b). Multivariable Cox analyses were performed to show PRACME’s independence in prognosis adjusting for other clinical variables (Fig. 4c). However, in LUSC patients, PRACME signatures were not informative of the patient prognosis (Supplementary Fig. 1).

### PRACME and ORACLE signatures are correlated and prognostic in both MPM and LUAD

**Fig. 5:**
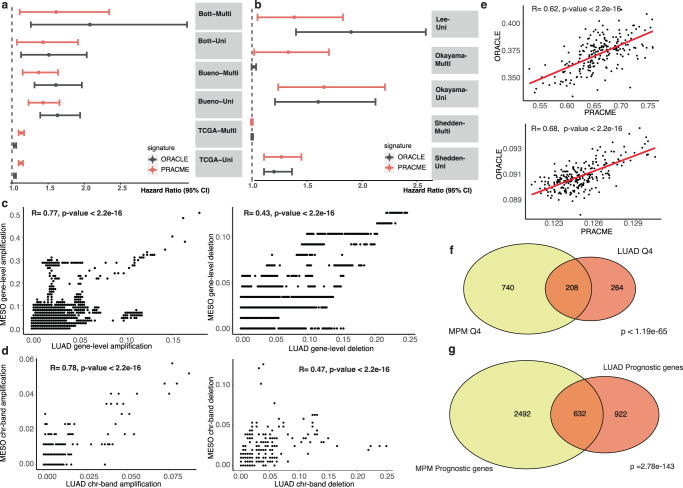
PRACME and ORACLE signatures are correlated and prognostic in both MPM and LUAD. **a** Univariable and Multivariable Cox regression models on Bueno, Bott and TCGA MPM datasets. **b** Univariable and Multivariable Cox regression models on Lee, Okayama and Shedden datasets; Each segment represents hazard ratio and 95-percent confidence interval. Red indicates PRACME, black indicates ORACLE. Vertical dash-line represents hazard ratio of 1.0. **c** Gene-level copy number variation correlation with amplification frequency on the left and deletion frequency on the right. *X*-axis represents LUAD gene-level CNV and *y*-axis represents MESO gene-level CNV. **d** Chromosome-band level CNV correlation with amplification frequency on the left panel and deletion frequency on the right panel. **e** Correlation of PRACME and ORACLE in Bueno MPM patients (top) and Okayama LUAD patients (bottom). **f** Overlapping Q4 genes in MESO (yellow) and in LUAD (red). **g** Overlapping prognostic genes in TCGA-MESO (yellow) and TCGA-LUAD (red).

To understand the cross-predictivity of ORACLE and PRACME in the two cancer types, we examined the correlation between their signature scores in both MPM and LUAD data (Fig. 5a, b). As shown in Fig. 5e, when applied to the Bueno data (MPM), the two signatures showed a high correlation (*R* = 0.62, *P* < 2.2e−16) across all samples. Similarly, two signatures showed strong correlation (*R* = 0.68, *P* < 2.2e−16) when applying to Okayama data (LUAD). To further understand why their signature scores are correlated, we compared the Q4 genes identified in MPM and LUAD, which were used for defining the two signatures. Out of the 948 MPM and 472 LUAD Q4 genes, 208 genes were shared between the two cancer types, which was significantly more than what is expected by chance (*P* < 2e−65). Among 7064 common genes shared between multiregional MPM data and TRACERx LUAD data, 948 genes belonged to Q4 in MPM and 472 genes belong to LUAD Q4; there were 208 overlapping Q4 genes, indicating that the Q4 genes were statistically overlapped with a significant *p*-value less than 1.19e−65. Similarly, we validated the similarity between two sets of prognostic genes in LUAD and MPM (*P* < 2.78e−143, Fig. 5g). In this way, we concluded that there remained high overlaps between prognostic genes in MPM and LUAD, and between Q4 genes in MPM and LUAD.

Since clonal genes tend to be located in amplified chromosomal regions, we then examined the similarity between LUAD and MPM in their copy number variation profiles. Based on the TCGA-LUAD and TCGA-MESO copy number variation data, we calculated the amplification and deletion frequency of all genes in the two cancer types. Interestingly, we found that both the gene amplification and deletion profiles were significantly correlated, but the former (*R* = 0.77, *P* < 2.2e−16) has a higher correlation than the latter (*R* = 0.43, *P* < 2.2e−16). A similar pattern was also observed on the chromosome band level, with amplification profiles (*R* = 0.78, *P* < 2.2e−16) more significantly correlated than deletion profiles (*R* = 0.47, *P* < 2.2e−16). Taken together, our results indicated the high correlation from many aspects may explain the cross-prediction of ORACLE and PRACME in two cancer types.

## Discussion

In this study, we generated multi-regional MPM RNA-seq data to define the PRACME clonal gene signature. We assessed and showed the prognostic values of this signature in two independent MPM datasets. Our results validated the strategy of selecting clonal genes with high intertumor but low intratumor heterogeneity for prognostic prediction, which was first proposed by Biswas et al in NSCLC. Prognostic signatures based on these genes are robust against the sampling bias issue and therefore more likely to be reproducible than conventional gene signatures developed without considering RNA-ITH. MPM and lung cancer are susceptible to sample bias due to their high ITH levels. Together with the ORACLE study, our analyses demonstrated the value of multiregional data in translational studies, especially in the field of biomarker development.

Interestingly, we found that the PRACME and ORACLE signatures can cross-predict patients’ prognosis in both MPM and LUAD, although they were originally developed from cancer-type specific data. Further, the cross-cancer prediction capability can be at least partially explained by the fact that the two cancer types shared many Q4 genes and similar gene amplification profiles. Of note, the gene amplification profiles are more correlated between MPM and LUAD than their deletion profiles. This is consistent with the observation that clonal genes are more likely to be located in amplified genomic regions presenting in dominant tumor clones. Due to their clonal nature, these genes tend to have lower intratumor heterogeneity at the transcriptomic level and therefore enriched in Q4 genes. This also explains the large number of shared Q4 genes between the two cancer types. Although MPM and LUAD are both thoracic cancers, it should be noted that they are quite different cancer types.

In this study, we showed that some gene signatures can predict prognosis in distinct but related cancer types. As such, it is possible to define prognostic signatures for rare cancers with limited data by leveraging larger data from a related cancer type. Our analysis indicated that, at least in MPM and LUAD, clonal gene signatures are likely to have cross-cancer predictive ability. However, it remains a challenge to utilize more broadly cross-cancer signatures in clinical application, because further research is needed to identify which cancer types are likely to share prognostic signatures and to what extent can the cross-prediction ability of clonal gene signature be extended to other cancer types.

We observed that the NSCLC signature ORACLE could actually predict MPM patient prognosis with a slightly higher performance than PRACME, which was specifically defined from MPM data. This result might be explained by the sample size difference of the data used for defining ORACLE and PRACME signatures. For ORACLE, Q4 genes were identified based on the TRACERx multi-regional data consisting of 89 samples from 28 patients and the prognostic genes were identified by using the TCGA-LUAD data consisting of 469 samples. In contrast, for PRACME, the Q4 were defined from 78 regions and 26 patients, and the prognostic genes were derived TCGA-MESO consisting of 87 patients. Both the multi-region data (for identifying Q4 genes) and the TCGA data (for identifying prognostic genes) have larger sample sizes for LUAD than MPM, which favors more accurate estimation of intratumor/intratumor heterogeneity of genes and a better list of prognostic genes in LUAD. Moreover, in our multi-regional data for MPM, the samples were collected from three regions including superior, lateral, and inferior. This approach is quite different from the method used by TRACERx for selecting samples from different regions of the same lung tumors. TRACERx cohort took whole-exome sequencing on primary tumor regions ranging from 2 to 8 and a median depth of 426x. The method used by TRACERX might be able to more effectively estimate the intratumor heterogeneity. Overall, this study improved our understanding of factors that determine clonal variables in both LUAD and MPM cancer evolution and provided a clonal gene signature with valuable risk prediction in patient survival. Overall, this study improved our understanding of factors that determine clonal variables in both LUAD and MPM cancer evolution and provided a clonal gene signature with valuable risk prediction in patient survival.

Our cross-prediction results showed the potential to define prognostic signatures for rare cancers and improved our understanding of common pathways among cancer types. Our signatures did not intersect with previous published 5-gene signature from Bai et al. and another 48-gene signature published recently by Nair et al.^19,26^. Further, our PRACME signature utilized multi-regional sampling to develop a robust clonal signature that provide prognostic values independently of stage and histology. Overall, this study improved our understanding of factors that determine clonal variables in both LUAD and MPM cancer evolution and provided a clonal gene signature with valuable risk prediction in patient survival.

## Methods

### Multi-regional MPM RNA-seq profiling and processing

This study was performed in accordance under Institutional Review Board protocol at Baylor College of Medicine (H-35782). We have complied with all relevant ethical regulations including the Declaration of Helsinki and written informed consent was obtained from all patients. Twenty six patients were those with a pathologic diagnosis of MPM undergoing macroscopic cytoreduction (MCR) by extrapleural pneumonectomy (EPP) or extended pleurectomy/decortication (eP/D) from 2017 to 2019. During the surgical procedure, we prospectively collected tumor samples from three different anatomical regions of the chest (superior, lateral, and inferior). Patient characteristics are described in Table 1.

**Table 1:** Characteristics of malignant pleural mesothelioma patients who underwent macroscopic complete resection

Variable	MPM
**Number of patients**	26
**Age**, yr. Median (range)	68.5 (39–80)
**Gender**	
Male	18 (69%)
Female	8 (31%)
**History of asbestos exposure (*n*** = **21)**	13 (62%)
**Histology**	
Epithelioid	17 (65%)
Biphasic	9 (35%)
**Clinical staging**	
I	12 (46%)
II	9 (35%)
III	5 (19%)
**Multimodality treatment**	
Neoadjuvant chemotherapy	9 (35%)
Adjuvant chemotherapy	7 (27%)
Adjuvant radiotherapy	6 (23%)
**Preoperative tumor PD-L1** > **1%**	14 (54%)
**Procedure**	
EPP	6 (23%)
P/D	20 (77%)

*EPP* extrapleural pneumonectomy, *MPM* malignant pleural mesothelioma, *P/D* pleurectomy/decortication, *PD-L1* programmed cell death 1 ligand 1.

To thoroughly investigate the complex heterogeneity within MPM, we systematically harvested tissue samples from the superior, lateral, and inferior regions during surgery. This strategic approach was based on the concept that different sections of the tumor were subject to diverse microenvironmental conditions and stress factors, potentially fostering distinct genetic and functional profiles. The three anatomical regions are selected as they best represent the heterogeneity of the pleural tissue. According to the 2023 NCCN guidelines, the recommended primary management of resectable MPM is surgical resection followed by chemotherapy, with additional consideration of sequential radiotherapy. Alternatively, induction chemotherapy using pemetrexed and cisplatin prior to surgical intervention is an option, followed by potential radiotherapy. In our cohort, 17 patients (65%) underwent primary surgery, while 9 patients (35%) received neoadjuvant chemotherapy before surgery. All tissue samples were collected during surgical procedures.

RNA was assessed for quality and quantified using an RNA 6000 Nano Lab Chip on a 2100 Bioanalyzer (Agilent Inc, Santa Clara, CA). We targeted 1.5 to 2 mg for RNASeq with RNA Integrity Number greater than 6. Libraries for RNA sequencing were sequenced with Illumina TruSeq Kits on a NovaSeq 6000 sequencer (Illumina Inc, San Diego, CA) to obtain 80 M reads per samle. Genome sequence GRCh38.p13 was used to map paired-end reads. The resulting mapped reads were assembled by STAR (ver.2.7.10b)^27^. Fragments per kilobase of exon per million fragments (FPKM) of the transcripts were used to evaluate mRNA expression levels^28^. Furthermore, we normalized all downstream analyses against the median of total read counts and used log_2_ transformation.

### Other data used in this study

The TCGA-LUAD (lung adenocarcinoma, consisting of 469 stage I and II samples) and the TCGA-MESO (consisting of 87 samples) RNA-seq data were downloaded from FireBrowse (http://firebrowse.org)^22,29^. The expression level of the genes was normalized and represented as the RSEM (RNA-Seq by Expectation-Maximization) values^28^. The Bueno MPM RNA-seq data were downloaded as raw fastq files from European Genomephenome Archive with accession number EGAS00001001563^30^. The data contained gene expression profiles for a total of 211 MPM samples. We gained corresponding clinical information and somatic mutations from whole-exome sequencing. Particular targeted mutations were retrieved from the supplementary materials from Bueno publication. The Bott data was downloaded from the Gene Expression Omnibus (GEO) database with the accession ID GSE29354^31^. The data contained the expression profiles for 53 MPM samples measured by Affymetrix microarrays. The clinical and pathological information about these samples were extracted from the supplementary documents of the original publication. In addition to the MPM datasets, we have downloaded 3 independent lung cancer datasets including both LUAD and LUSC (lung squamous cell carcinoma) patients. Microarray data and clinical data were downloaded from the Gene expression Omnibus for 226 LUAD patients enrolled by Okayama et al (GSE31210), 442 LUAD patients enrolled by Shedden et al (GSE68465), 63 LUAD patients enrolled by Lee et al (GSE8894)^32–34^. Further, we downloaded expression profiling of 249 LUSC patients from Bueno et al (GSE157011)^35^. We used paired-end fasq files with trimmed adapters for downloaded RNA-seq dataset. HISAT2 was used to align human genome assembly GRCh38 and SAMtools^36^. We used HTSeq to calculate read counts per sample^37^.

### Defining PRACME gene signatures

#### Intratumor heterogeneity and intertumor heterogeneity scores

For each gene, we calculated an intratumor heterogeneity score and an intertumor heterogeneity score^9^. To obtain the intratumor heterogeneity score, we first calculated the standard deviation of its expression values across the 3 regions for each patient, and then took the median standard deviation across all patients (n = 26). The intertumor heterogeneity score for a gene was calculated by randomly sampling one of the 3 regions from each patient and taking the standard deviation across the resulting single-region cohort. This procedure was repeated 100 times and then the average score across all iterations was used as the final intertumor heterogeneity score.

#### Clustering coefficient scores

We measured the proportion of patients with all regions in the same cluster against the number of clusters (2–26 clusters)^38^. For each gene, we ran a k-means algorithm based on the gene expression and calculated the percentage of patients with all tumor regions in the same hierarchical cluster. Next, we averaged the value to get the concordance coefficient for that gene. We manually set the cutoff as 0.2 and kept the genes that have a concordance coefficient greater than 0.2.

To define a clonal gene signature, we integrated the above-described multi-region MPM RNA-seq data and the TCGA-MESO RNA-seq data by following a similar pipeline used for defining ORACLE^9^. In short, the following major steps were performed. First, starting with 20,501 genes from the TCGA-MESO data, we filtered out 50% of the genes with low median expression values across all samples (10,250/20,501 genes). Second, prognostic genes (1667/10,250 genes) were identified as those correlated with overall survival (*P* < 0.01) in the TCGA-MESO data according to univariable Cox regression analysis. Third, from these prognostic genes, we identified Q4 genes with high intertumor but low intratumor heterogeneity scores calculated from the multi-regional MPM RNA-seq data (323/1,667 genes). Fourth, we selected the genes with clustering coefficient scores greater than 0.2 (118/323 genes). To reduce collinearity among candidate gene predictors, we used lasso Cox regression model. Finally, by applying the lasso Cox regression model with tuning parameter of 0.06 to the TCGA-MESO data, we identified a core set of 29 prognostic genes. After lasso selection, we fitted a final regression model to derive regression coefficients for the genes that were retained. These genes formed the final clonal gene signature named PRACME as listed in Supplemental Table 1.

### The formula to calculate sample-specific prognostic scores

For each patient, we calculated the patient-specific prognostic score by calculating the dot product of gene expression values and a gene-specific weight using the following formula:

$$S=\varSigma {c}_{i}{x}_{i}$$

in which ${x}_{i}$ is log transformed gene expression and ${c}_{i}$ is the coefficient from the lasso regression model for gene i.

### Statistical analysis

All statistical analyses were conducted in the R software. We used “coxph” function for univarible and multivarible Cox regression. In the multivariable analysis, we included the variables that were significantly associated with patient prognosis in univariable analysis and several established variables including sex and stage. R package “survival” was implemented to perform survival analyses. The “survdiff” function was used to compare patient groups using log-rank test; patients with overall survival less than 30 days were excluded for TCGA-MESO. The “wilcox.test” and the “t.test” functions were used to compare two groups of selected measurement. Package “VennDiagram” was used to show overlapping information between multiple groups. Function “phyper” was used to calculate enrichment ration and p-value based on hypergeometric test (“Fisher’s exact test”). Lasso regression was performed using the “glmnet” package applying penalty term^39^. Correlation analysis used function “cor.test” with default Spearman coefficients. Package “kmeans” was used to calculate clustering coefficient scores. Package “ggplot2” was used to generate boxplot and Kaplan-Meier survival time curves. A p-value of 0.05 was the significant threshold for statistical test, unless noted otherwise. We used the canonical pathways from BIOCARTA, KEGG^40^, PID^41^, and REACTOME^42^ databases by R package “gsva”^43^.

### Inferring immune cell infiltration

We used the previously developed computational algorithm to infer the selected immune cells’ infiltration level based on the gene expression profiles^44,45^. This algorithm estimates the immune cells’ infiltration levels by examining the expression levels of immune-cell-specific genes. Thus, we received the infiltration scores of six immune cell types including memory B cells, CD4+ T cells, CD8+ T cells, naïve B cells, NK cell and myeloid cells.

### Reporting summary

Further information on research design is available in the Nature Research Reporting Summary linked to this article.

### Supplementary information

Supplemental Material

REPORTING SUMMARY

## Data Availability

Multiregional MPM RNAseq data have been deposited into NCBI Gene Expression Omnibus (GSE247203).

## Code availability

The code to perform analysis and validation of the signature, and reproduce all figures in the manuscript can be accessed with no restrictions at https://github.com/YPeiLin/PRACME--NPJ-precision-oncology- Detailed packages included in the section Method: Statistical analysis including “coxph”, “survival”, “wilcox.test”, “Venndiagram”, “ggplot2” and “glmnet”.
