# Supplementary material for: Clonal gene signatures predict prognosis in mesothelioma and lung adenocarcinoma
Source: NPJ Precis Oncol. 2024 Feb 23;8:47. doi: 10.1038/s41698-024-00531-y (PMC10891127; doi:10.1038/s41698-024-00531-y)
Supplement: Supplementary file 1 — Supplemental Material [file 41698_2024_531_MOESM1_ESM.pdf]

Supplementary information

Supplementary Table 1 PRACME clonal gene signature
Supplementary Table 2 Univariable Cox Regression of PRACME and ORACLE in MPM data
Supplementary Table 3 Multivariable Cox Regression of PRACME and ORACLE in MPM data
Supplementary Table 4 Bueno MPM stratification analysis
Supplementary Table 5 Correlation between PRACME and Throsson-immunity table
Supplementary Table 6 Correlation between PRACME and GSVA scores
Supplementary Table 7 Summary of Cox Regression of PRACME on all clinical variables
Supplementary Table 8 Mutation status in MPM data

Supplementary Figure 1 Association of PRACME signature score with additional clinical features
Supplementary Figure 2 PRACME signature is not predictive of prognosis in lung squamous carcinoma
Supplementary Figure 3 PRACME signature is negatively correlated to immune infiltration levels
Supplementary Figure 4 PRACME score between histology in multiregional data

Supplementary Table 1 PRACME clonal gene signature

Gene Name	Coefficient	Location in genome
ACAT2	0.03	6q25.3
ADAM9	0.04	8p11.22
ADAMTS1	0.03	21q21.3
AGPAT5	0.09	8p23.1
B3GALNT2	0.23	1q42.3
COL11A1	0.02	1p21.1
CPT2	-0.52	1p32.3
DSC3	0.02	18q12.1
FAT1	0.12	4q35.2
FTSJD1	-0.01	16q22.2
KIAA1199	-0.02	15q25.1
MAP4K4	0.21	2q11.2
MCTP2	-0.27	15q26.2
METTL1	0.07	12q14.1
MR1	-0.27	1q25.3
NRIP1	0.2	21q11.2-q21.1
PANK4	-0.11	1p36.32
PLXND1	-0.02	3q22.1
PWP1	0.05	12q23.3
RAP1GDS1	0.65	4q23
RUVBL2	0.27	19q13.33
SH3TC1	0.5	4p16.1
SNX5	0.57	20p11.23
SPAG5	0.34	17q11.2
SPRY2	-0.24	13q31.1
SYS1	-0.11	20q13.12
TMEM183A	0.24	1q32.1
TSPAN3	0.15	15q24.3
UVRAG	-0.57	11q13.5

Supplementary Table 2 Univariable Cox Regression

	Univariable Cox Regression		
	TCGA-MESO	Bueno	Bott_GSE29354
PRACME	1.12(2.6e-16)	1.40 (4.6e-04)	1.61 (1.3e-02)
ORACLE	1.04 (1.0e-08)	1.63 (1.6e-09)	1.51 (6.2e-03)

Supplementary Table 3 Multivariable Cox Regression

	Multivariable Cox Regression		
	TCGA-MESO	Bueno	Bott_GSE29354
PRACME	1.13(5.3e-13)	1.37 (4.60e-04)	1.61(1.3e-02)
ORACLE	1.04(4.0e-07)	1.61 (4.46e-06)	2.08(4.1e-03)

Supplementary Table 4 Bueno MPM stratification

**Validation Histology**	**HR**	**p-value**
Biphasic (n = 62)	1.03	0.88
Epithelioid (n = 141)	1.61	7.21e-07
All patients (n = 211)	1.43	2.63e-06
**Patient Stages**	**HR**	**p-value**
Early (stage I and II) (n=58)	1.49	0.02
Late (stage III and IV) (n=125)	1.45	4.04e-05
**Smoking History**	**HR**	**p-value**
Smoking history (n = 114)	1.48	3.64e-08
No smoking history (n = 96)	1.41	1.14e-03

Supplementary Table 5 Correlation between PRACME score and Throsson-immunity table

Intratumor Heterogeneity	0.40
Proliferation	0.77
TGF beta Response	0.47

Supplementary Table 6. Correlation with GSVA pathways

Pathways	PRACME
REACTOME_PHASE1_FUNCTIONALIZATION_OF_COMPOUNDS	0.67
REACTOME_NUCLEAR_SIGNALING_BY_ERBB4	0.59
KEGG_LINOLEIC_ACID_METABOLISM	0.55
REACTOME_BIOLOGICAL_OXIDATIONS	0.58
REACTOME_COMPLEMENT_CASCADE	0.52

KEGG_DRUG_METABOLISM_CYTOCHROME_P450	0.52
REACTOME_BILE_ACID_AND_BILE_SALT_METABOLISM	0.48
KEGG_TYROSINE_METABOLISM	0.48
REACTOME_CYTOCHROME_P450_ARRANGED_BY_SUBSTRATE_TYPE	0.52

Supplementary Table 7 Summary of Cox Regression of PRACME on all clinical variables

Bueno Univariable analysis with PRACME

		**HR**	**p-value**
Age	Age	1.0	0.008
Stage	Stage I<br>Stage II<br>Stage III<br>Stage IV	reference<br>1.8<br>2.1<br>2.5	reference<br>0.2<br>0.1<br>0.06
Sex	Female<br>Male	reference<br>1.3	reference<br>0.2
Histology	Epithelioid<br>Biphasic<br>Sacomatoid	reference<br>1.5<br>2.1	reference<br>0.01<br>0.07
Asbestos exposure	No<br>Yes	reference<br>1.9	reference<br>0.001

Bott Univariable Analysis with PRACME

		**HR**	**p-value**
Age	Age	1.0	0.04
Stage	Stage I<br>Stage II<br>Stage III<br>Stage IV	reference<br>1.5<br>3.7<br>4.8	reference<br>0.5<br>0.03<br>0.01
Sex	Female<br>Male	reference<br>1.1	reference<br>0.8
Histology	Epithelioid<br>Biphasic<br>Sacomatoid	reference<br>1.2<br>1.5	reference<br>0.002<br>0.007
Asbestos exposure	No<br>Yes	reference<br>1.0	reference<br>0.9

Supplementary Table 8 Mutation status in MPM data

Mutation	Mutated Size	T-score	T-test p-value	Wilcox Test p-value	Mutated Mean	WO Mut Mean
NF2	37	2.80	0.007	0.002	0.66	0.65
BAP1	46	0.94	0.35	0.35	0.67	0.65
SETD2	17	-1.40	0.18	0.18	0.64	0.66
TP53	16	0.24	0.81	0.94	0.66	0.65

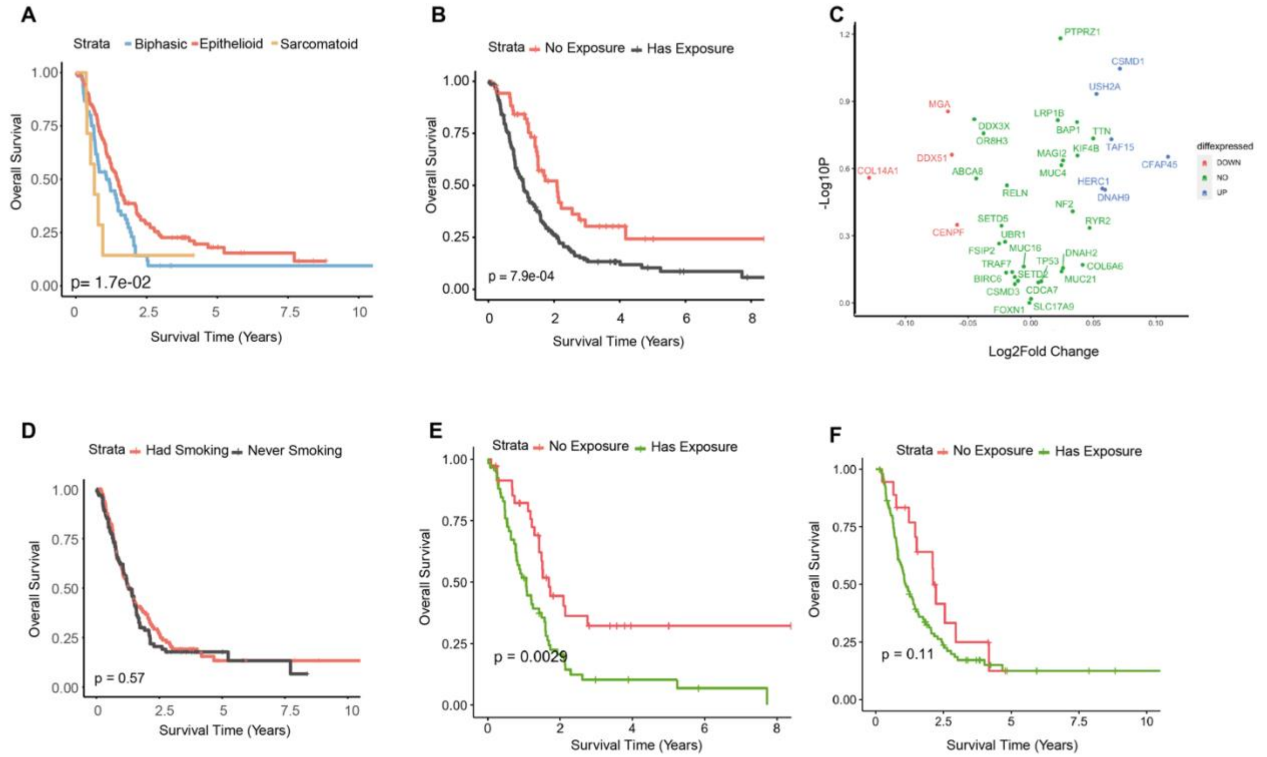

Supplementary Figure 1 Association of PRACME signature score with additional clinical features

In Bueno MPM dataset, A) PRACME score for three histology; B) PRACME for asbestos exposure; C) Targeted mutation volcano plot; D) PRACME score for patients smoking history. We separated patients with never smokers and ever smokers; E) PRACME score for asbestos exposure in never-smokers; F) PRACME score for asbestos exposure in ever-smokers.

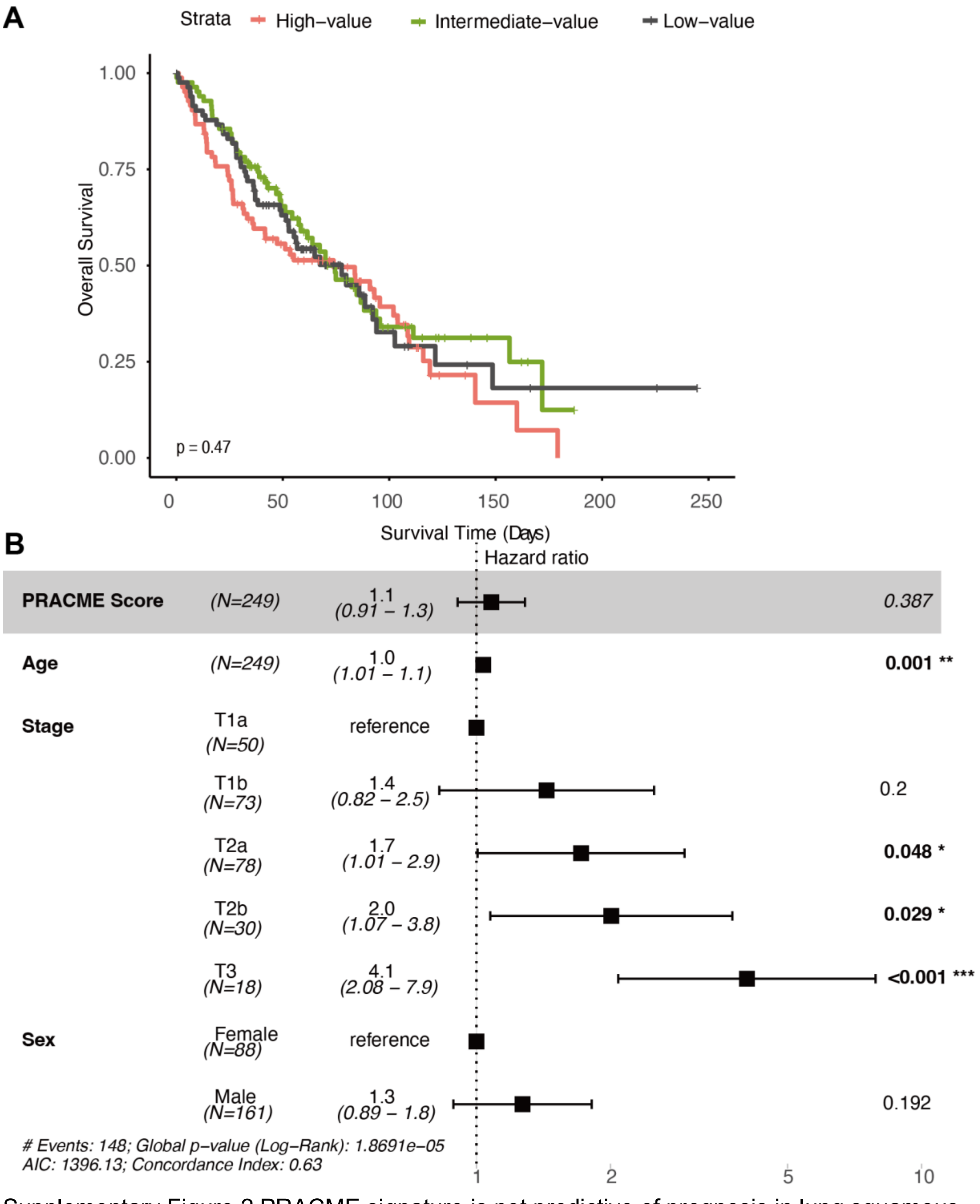

Supplementary Figure 2 PRACME signature is not predictive of prognosis in lung squamous carcinoma

In Bueno GSE157011 LUSC patients, A) PRACME score for high-, intermediate-, and low-group patients; B) PRACME score adjusted for age, stage, and sex.

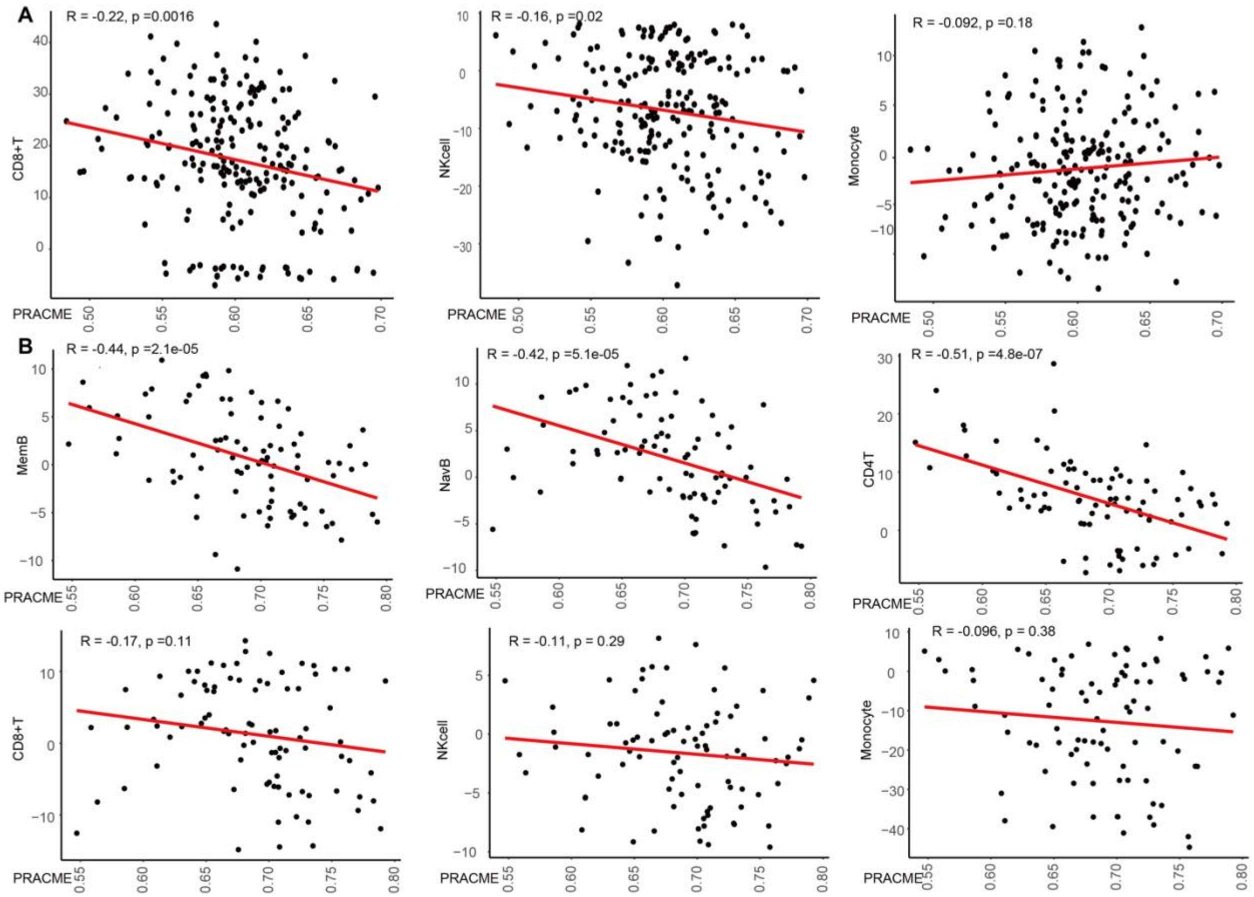

Supplementary Figure 3 PRACME signature is negatively correlated to immune infiltration levels In Bueno MPM dataset, A) PRACME correlation with CD8+T cells, NK cells and Monocyte infiltration. In TCGA-MESO, B) PRACME correlation with Memory B cells, Naïve B cells, CD4+T cells, CD8+T cells, NK cells and Monocyte infiltration.

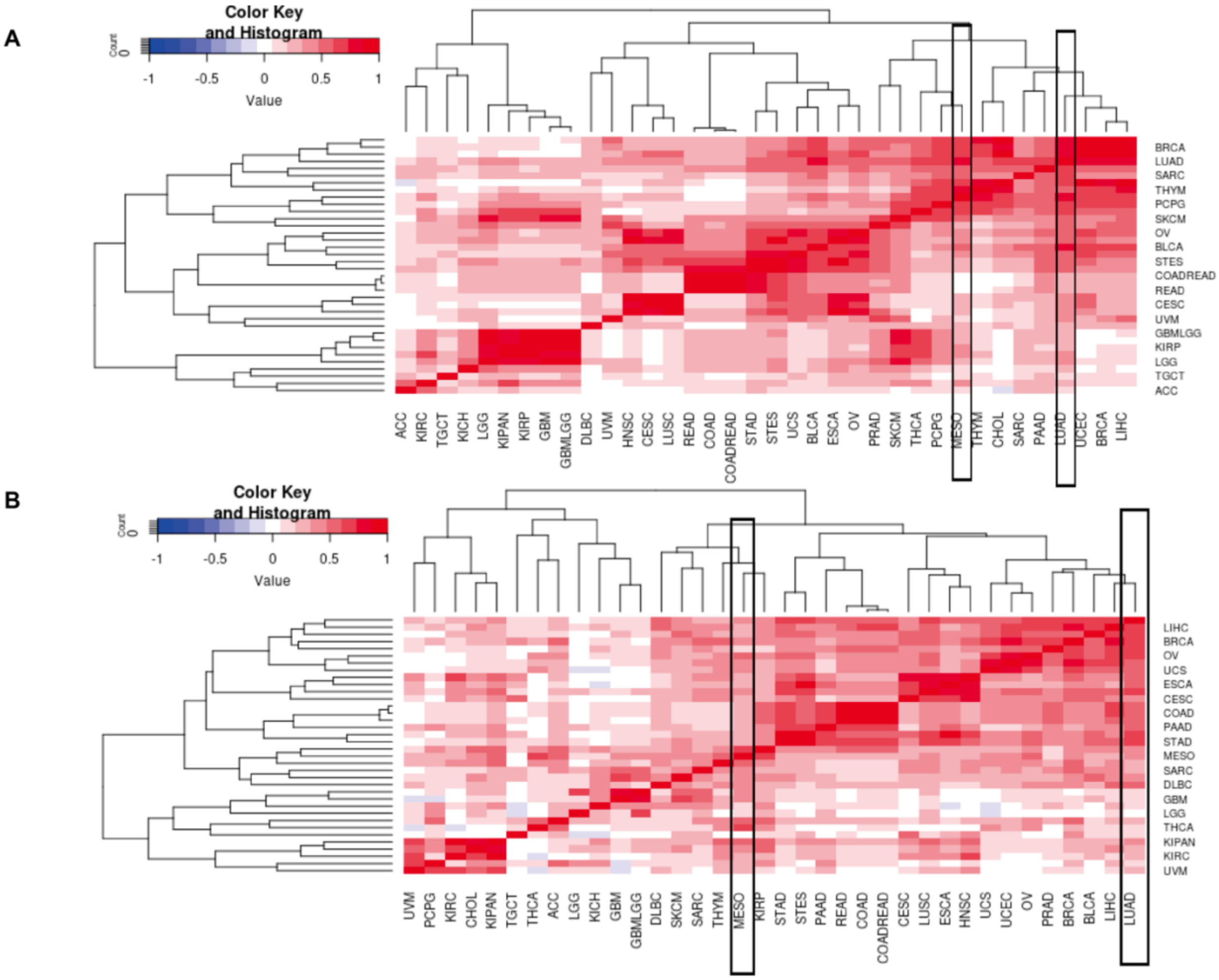

In TCGA-MESO, A) Gene-level amplification frequency correlation between 37 other common cancer types; B) Gene-level deletion frequency correlation between 37 other common cancer types

Supplementary Figure 4 Wilcox Test Between signature score and histology in multiregional data

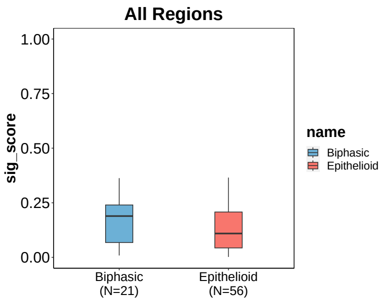

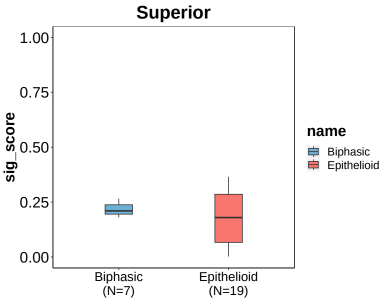

p-value = 0.22

p-value = 0.9

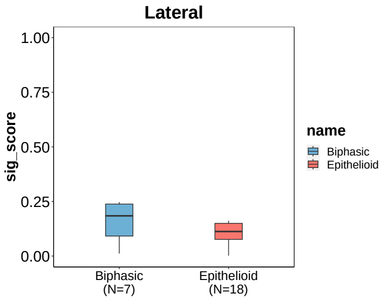

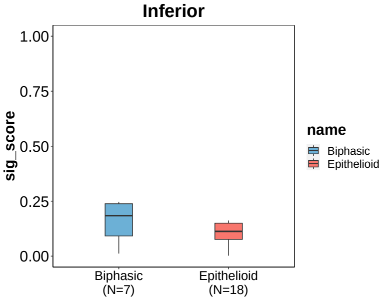

p-value = 0.7

p-value = 0.06
