# Supplementary material for: Clonal gene signatures predict prognosis in mesothelioma and lung adenocarcinoma
Source: NPJ Precis Oncol. 2024 Feb 23;8:47. doi: 10.1038/s41698-024-00531-y (PMC10891127; doi:10.1038/s41698-024-00531-y)
Supplement: Supplementary file 2 — REPORTING SUMMARY [file 41698_2024_531_MOESM2_ESM.pdf]

nature portfolio

Corresponding author(s): Chao Cheng, Christopher I. Amos

Last updated by author(s): Jan 11, 2024

Reporting Summary

Nature Portfolio wishes to improve the reproducibility of the work that we publish. This form provides structure for consistency and transparency in reporting. For further information on Nature Portfolio policies, see our Editorial Policies and the Editorial Policy Checklist.

Statistics

For all statistical analyses, confirm that the following items are present in the figure legend, table legend, main text, or Methods section.

n/a	Confirmed	
☐	☒	The exact sample size (*n*) for each experimental group/condition, given as a discrete number and unit of measurement
☐	☒	A statement on whether measurements were taken from distinct samples or whether the same sample was measured repeatedly
☐	☒	The statistical test(s) used AND whether they are one- or two-sided<br>*Only common tests should be described solely by name; describe more complex techniques in the Methods section.*
☐	☒	A description of all covariates tested
☐	☒	A description of any assumptions or corrections, such as tests of normality and adjustment for multiple comparisons
☐	☒	A full description of the statistical parameters including central tendency (e.g. means) or other basic estimates (e.g. regression coefficient) AND variation (e.g. standard deviation) or associated estimates of uncertainty (e.g. confidence intervals)
☐	☒	For null hypothesis testing, the test statistic (e.g. *F*, *t*, *r*) with confidence intervals, effect sizes, degrees of freedom and *P* value noted<br>*Give P values as exact values whenever suitable.*
☒	☐	For Bayesian analysis, information on the choice of priors and Markov chain Monte Carlo settings
☒	☐	For hierarchical and complex designs, identification of the appropriate level for tests and full reporting of outcomes
☐	☒	Estimates of effect sizes (e.g. Cohen's *d*, Pearson's *r*), indicating how they were calculated

Our web collection on statistics for biologists contains articles on many of the points above.

Software and code

Policy information about availability of computer code

Data collection	RNA was assessed for quality and quantified using an RNA 6000 Nano Lab Chip on a 2100 Bioanalyzer (Agilent Inc, Santa Clara, CA). Libraries for RNA sequencing were sequenced with Illumina TruSeq Kits on a NovaSeq 6000 sequencer (Illumina Inc, San Diego, CA) to obtain 80M reads per sample. Genome sequence GRCh38.p13 was used to map paired-end reads. The resulting mapped reads were assembled by STAR (ver.2.7.10b). Fragments per kilobase of exon per million fragments (FPKM) of the transcripts were used to evaluate mRNA expression levels.
Data analysis	Detailed packages included in the section Method: Statistical analysis including "coxph", "survival", "wilcox.test", "Venndiagram", "ggplot2" and "glmnet".

For manuscripts utilizing custom algorithms or software that are central to the research but not yet described in published literature, software must be made available to editors and reviewers. We strongly encourage code deposition in a community repository (e.g. GitHub). See the Nature Portfolio guidelines for submitting code & software for further information.

Data

Policy information about availability of data

All manuscripts must include a data availability statement. This statement should provide the following information, where applicable:

- Accession codes, unique identifiers, or web links for publicly available datasets
- A description of any restrictions on data availability
- For clinical datasets or third party data, please ensure that the statement adheres to our policy

Multi-regional Mesothelioma data uploaded Gene Expression Omnibus with accession number is GSE247203.

Research involving human participants, their data, or biological material

Policy information about studies with human participants or human data. See also policy information about sex, gender (identity/presentation), and sexual orientation and race, ethnicity and racism.

Reporting on sex and gender	Sex and gender information about 26 patients with malignant pleural mesothelioma who underwent macroscopic complete resection was described in Table 1.
Reporting on race, ethnicity, or other socially relevant groupings	The distribution of ethnic and racial categories mirrors the demographic data in our center, i.e. approximately 90% White subjects (including 5% Hispanic subjects), 5% Black or African American subjects, and 3% Asian subjects. The other minority groups account for 2% of our patient population. Multivariable analysis including confounders was performed.
Population characteristics	Patient characteristics is described in Table 1. Median age was 68.5 years old, and 18 males (69%) were included. 17 patients (65%) underwent primary surgery, while 9 patients (35%) received neoadjuvant chemotherapy before surgery. All tissue samples were collected during surgical procedures, which means that 65% of the samples came from treatment-naïve patients, and 35% from those who had preoperative chemotherapy.
Recruitment	This retrospective study used our bio-repository protocol. Twenty six patients were those with a pathologic diagnosis of MPM undergoing consecutive MCR by extrapleural pneumonectomy (EPP) or pleurectomy/decortication (P/D) from 2017 to 2019.
Ethics oversight	Baylor College of Medicine

Note that full information on the approval of the study protocol must also be provided in the manuscript.

Field-specific reporting

Please select the one below that is the best fit for your research. If you are not sure, read the appropriate sections before making your selection.

☒ Life sciences ☐ Behavioural & social sciences ☐ Ecological, evolutionary & environmental sciences

For a reference copy of the document with all sections, see nature.com/documents/nr-reporting-summary-flat.pdf

Life sciences study design

All studies must disclose on these points even when the disclosure is negative.

Sample size	As a retrospective tissue collection study, we generated a high-quality multi-regional RNA-seq expression dataset including 78 samples collected from 26 MPM patients, each having 3 regions (superior, lateral and inferior) of the same tumor.
Data exclusions	No data were excluded from the analyses.
Replication	To thoroughly investigate the complex heterogeneity within MPM, we systematically harvested tissue samples from the superior, lateral, and inferior regions during macroscopic complete resection. This strategic approach was based on the concept that different sections of the tumor were subject to diverse microenvironmental conditions and stress factors, potentially fostering distinct genetic and functional profiles.
Randomization	Allocation was not random. Multivariable analysis including confounders was performed.
Blinding	Blinding was not relevant to the study since this study was a retrospective study, in which the tumor samples were consequently collected.

Reporting for specific materials, systems and methods

We require information from authors about some types of materials, experimental systems and methods used in many studies. Here, indicate whether each material, system or method listed is relevant to your study. If you are not sure if a list item applies to your research, read the appropriate section before selecting a response.

Materials & experimental systems

n/a	Involved in the study
☒	☐ Antibodies
☒	☐ Eukaryotic cell lines
☒	☐ Palaeontology and archaeology
☒	☐ Animals and other organisms
☐	☒ Clinical data
☒	☐ Dual use research of concern
☒	☐ Plants

Methods

n/a	Involved in the study
☒	☐ ChIP-seq
☒	☐ Flow cytometry
☒	☐ MRI-based neuroimaging

Clinical data

Policy information about clinical studies

All manuscripts should comply with the ICMJE guidelines for publication of clinical research and a completed CONSORT checklist must be included with all submissions.

Clinical trial registration

Institutional Review Board protocol at Baylor College of Medicine (H-35782).

Study protocol

The immune landscape of thoracic malignancies

Data collection

Twenty six patients were those with a pathologic diagnosis of malignant pleural mesothelioma (MPM) undergoing macroscopic complete resection (MCR) by extrapleural pneumonectomy (EPP) or pleurectomy/decortication (P/D) from 2017 to 2019. To thoroughly investigate the complex heterogeneity within MPM, we systematically harvested tissue samples from the superior, lateral, and inferior regions during MCR.

Outcomes

Intratumor heterogeneity and Intertumor heterogeneity scores: For each gene, we calculated an intratumor heterogeneity score and an intertumor heterogeneity score. To obtain the intratumor heterogeneity score, we first calculated the standard deviation of its expression values across the 3 regions for each patient, and then took the median standard deviation across all patients (n=26). The intertumor heterogeneity score for a gene was calculated by randomly sampling one of the 3 regions from each patient and taking the standard deviation across the resulting single-region cohort. This procedure was repeated 100 times and then the average score across all iterations was used as the final intertumor heterogeneity score.

Plants

Seed stocks

Report on the source of all seed stocks or other plant material used. If applicable, state the seed stock centre and catalogue number. If plant specimens were collected from the field, describe the collection location, date and sampling procedures.

Novel plant genotypes

Describe the methods by which all novel plant genotypes were produced. This includes those generated by transgenic approaches, gene editing, chemical/radiation-based mutagenesis and hybridization. For transgenic lines, describe the transformation method, the number of independent lines analyzed and the generation upon which experiments were performed. For gene-edited lines, describe the editor used, the endogenous sequence targeted for editing, the targeting guide RNA sequence (if applicable) and how the editor was applied.

Authentication

Describe any authentication procedures for each seed stock used or novel genotype generated. Describe any experiments used to assess the effect of a mutation and, where applicable, how potential secondary effects (e.g. second site T-DNA insertions, mosiacism, off-target gene editing) were examined.
